# Micronutrient Supplement Use and Diet Quality in University Students

**DOI:** 10.3390/nu7021094

**Published:** 2015-02-05

**Authors:** Adam R. Wiltgren, Alison O. Booth, Gunveen Kaur, Sara Cicerale, Kathleen E. Lacy, Maree G. Thorpe, Russell S. J. Keast, Lynn J. Riddell

**Affiliations:** 1 Centre for Physical Activity and Nutrition Research, School of Exercise and Nutrition Sciences, Deakin University, 221 Burwood Hwy, Burwood, Victoria 3125, Australia; E-Mails: arwil@deakin.edu.au (A.R.W.); alison.booth@deakin.edu.au (A.O.B.); gunveen.kaur@deakin.edu.au (G.K.); katie.lacy@deakin.edu.au (K.E.L.); maree.thorpe@deakin.edu.au (M.T.); 2 Centre for Advanced Sensory Science, School of Exercise and Nutrition Sciences, Deakin University, 221 Burwood Hwy, Burwood, Victoria 3125, Australia; E-Mails: sara.cicerale@deakin.edu.au (S.C.); Russell.keast@deakin.edu.au (R.S.J.K.)

**Keywords:** micronutrient supplements, diet quality, university students, dietary guidelines

## Abstract

Many national and international public health organisations recommend achieving nutrient adequacy through consumption of a wide variety of nutritious foods. Despite this, dietary supplement sales continue to increase. Understanding the characteristics of micronutrient supplement users and the relationship with diet quality can help develop effective public health interventions to reduce unnecessary consumption of vitamin and mineral supplements. Participants (*n* = 1306) were a convenience sample of students studying first year food and nutrition. Data was collected via a Food and Diet Questionnaire (FDQ) and a Food Frequency Questionnaire (FFQ). Supplement users were defined as participants who indicated consuming any listed supplement as frequently as once a month or more. Diet quality was assessed using a Dietary Guideline Index (DGI) score. Prevalence of supplement use was high in this study population with 56% of participants reporting supplement use; the most popular supplements consumed were multivitamins (28%) and vitamin C (28%). A higher DGI score was significantly associated with an increased likelihood of supplement use (mean: 105 ± 18 *vs*. 109 ± 17, *p* = 0.001). Micronutrient supplement use was associated with a higher DGI score, suggesting that supplements are more likely to be used by those who are less likely to require them.

## 1. Introduction

Food and nutrient guidelines are established by national health agencies to provide members of the public and health practitioners with evidence based recommendations on nutrient and dietary intakes that are associated with low risk of nutrient deficiencies and diet related chronic diseases [1,2]. The Australian Dietary Guidelines, World Cancer Research Fund/American Institute of Cancer Research and the World Health Organisation recommend meeting nutrient needs through consuming a wide variety of nutritious foods via a mixed and balanced diet [2,3,4]. In Australia, iodine and folate are the only micronutrient supplements recommended by national health agencies and these recommendations are specific for pregnant women, women planning a pregnancy or breastfeeding women [5,6].

Despite these public health recommendations, the Australian dietary supplement industry has seen a significant increase in sales over the last few years [7]. From 2007 to 2012 vitamin sales grew 34% to reach a value of AUD $527 million and mineral supplement sales grew 49% to reach a value of AUD $119 million [7]. Multivitamins are the most popular supplement sold and the turnover in 2012 was AUS $328 million [7].

Understanding the demographic characteristics of those that use supplements, the nature of supplement use, and the implications of use on dietary intakes can help develop effective public health interventions to reduce the consumption of unnecessary and costly micronutrient supplement use [1]. Large cross-sectional population studies detailing micronutrient supplement use for different population groups in Australia are relatively scarce. Data on micronutrient supplement use were collected as part of the recent 2011–2012 National Nutrition and Physical Activity Survey (NNPAS) but is yet to be published in full [8]. Thus, the 2007 Australian National Children’s Nutrition and Physical Activity Survey (ANCNPAS) is the most recent population data on supplement use for Australia but this survey was limited to 2–16 year olds [9]. Large population data are more extensive for the US and provide a platform for understanding dietary supplement use [10,11,12,13]. The findings from these combined population based studies indicate that supplement use is higher for females, Caucasians and Asians, those with a higher income and education level, those with a healthy BMI and those that consider themselves having general good health [8,9,10,12,14]. Supplement use also increases with age after adolescence, with young adolescents the least likely to consume supplements [8,10]. Of the few studies available, micronutrient supplement users were more likely to have an adequate nutrient intake from food alone [13,15,16,17].

Meeting nutrient needs from diet alone may be difficult for individuals with increased requirements or inadequate intakes due to dietary restrictions or intolerances [2]. Micronutrient supplements can therefore be of benefit for these at-risk groups [18,19,20,21]. However, there is evidence suggesting that dietary supplements have no benefit for a healthy individual and excessive intakes of micronutrients can cause adverse health effects [1,2,22,23,24,25]. Clinical issues associated with excess intake of nutrients are nearly always linked with intakes of dietary supplements [1]. Understanding the link between diet quality and dietary supplement use is important to determine the efficacy of supplement practices. It is important to find a balance between excessive and inadequate nutrient intakes.

There is limited information regarding the characteristics of micronutrient supplement users within Australia and limited research exploring the relationship between diet quality and micronutrient supplement use. The present study aims to investigate demographic characteristics, diet quality and micronutrient supplement use in a sample of students studying food and nutrition.

## 2. Materials and Methods

### 2.1. Participants and Recruitment

Participants were recruited from a convenience sample of students enrolled in a first-year food and nutrition unit during 2011 to 2013 at Deakin University, 1408 of the 1603 enrolled in the unit consented to take part in the study (88% response rate). The completion of the questionnaires is part of an assessment task and the completion of this unit is compulsory for those obtaining a human nutrition degree, however other health related degrees also complete this unit as an elective. Participants who completed only one out of two questionnaires (*n* = 42) or had misreported their student identification numbers which resulted in being unable to link the two questionnaires (*n* = 60) were removed from the data set. Therefore, a total of 1306 participants (81%) were incorporated in final analysis.

### 2.2. Ethics

All procedures were approved by Deakin University’s Human Research Ethics Committee on 7/12/2009 (Ethics number: EC2009-163).

### 2.3. Food and Diet Questionnaire (FDQ)

The self-administered Food and Diet Questionnaire (FDQ) has been used previously [26,27] and was based on questionnaires used in studies investigating the eating habits of young adults [28,29]. The FDQ contained questions related to general demographic and anthropometric information (sex, age, nationality, maternal education level, height (cm) and weight (kg)) as well as questions related to dietary behaviours, opinions and influences (smoking behaviours, alcohol consumption, perceived healthiness of their own diet, consumption of specific diets with options such as vegetarian, vegan, lactose free, gluten free, low fat, diabetic, low fat/low sugar and yeast free, and sources of food, nutrition and health information).

### 2.4. Food Frequency Questionnaire (FFQ)

The 107 item Food Frequency Questionnaire (FFQ) has previously been used in the 1995 National Nutrition Survey and validated for Australian populations [30,31,32]. Section one of the FFQ contained questions relating to the consumption frequency of eight food and beverage groups as well as micronutrient supplements over the past month. Participants were asked to recall the frequency of consumption of these food groups in the past month, and were given nine possible frequency options ranging from “Never, or less than once a month” to “6+ times per day”. Section two of the FFQ contained further questions related to the frequency of particular food habits such as consuming low-fat dairy alternatives, trimming the fat from meat or the adding of salt to foods.

### 2.5. Diet Quality Assessment

To assess diet quality, a previously developed Dietary Guideline Index (DGI) was used [33]. Dietary information collected from the FFQ was used to assess the diet quality using a 150-point quality index for each participant. The DGI is comprised of fifteen components with each component having a maximum possible score of 10 points, a higher DGI score reflects a better diet quality [33]. The fifteen components of the DGI are set to assess a participant’s intake of key nutrients from core food groups, the proportion of key nutrient intakes from healthy food types (e.g., lean meats or wholegrain cereals), variety of foods in the diet and intakes of unhealthy foods. Those that reported to be in between the criteria for minimum and maximum had scores proportionately adjusted; for example if a participant reporting consuming one serve of fruit (half the recommended amount as per the 2005 dietary guidelines [34]) they received a score of 5 for that component—half of the maximum possible score. This method of diet quality assessment has been previously validated [33]; a higher DGI score has shown to be inversely related with poor health outcomes in previous research [27,33,35].

The DGI reflects the age and sex specific recommendations from the 2005 Food for Health guidelines within the Australian Dietary Guidelines [33,34]. The 2013 Australian Dietary Guidelines [2] were not used to assess diet quality in the current study as 2011 and 2012 data were collected prior to the release of the new guidelines. The 2013 data was collected in March 2013, one month after the release of the 2013 guidelines, and this was viewed as an insufficient time period to affect dietary patterns of the 2013 cohort, therefore the 2005 guidelines were considered more appropriate for calculating the DGI score.

### 2.6. Micronutrient Supplement Use Assessment

Participants were asked to record in the FFQ what micronutrient supplements they consumed (if any) and the frequency of use. The micronutrient supplement types that were assessed were limited to those included in the FFQ; multivitamin with iron or other mineral, multivitamin, vitamin A, vitamin B, vitamin C, vitamin E, β-carotene, calcium, folate/folic acid, iron and zinc. A micronutrient supplement user was defined as anyone that reported using a micronutrient supplement as frequently as once a month or more over the past month within the FFQ.

### 2.7. Statistical Analysis 

Data analysis was conducted using SPSS version 22.0 by IBM. Descriptive statistics (mean, standard deviation and quartiles) were used to describe continuous variables such as age, BMI and DGI results. Frequency statistics (number and percentage) were used to describe categorical variables. Analyses of results across the three years of data collection were conducted and no significant differences were observed, thus the data set was treated as one sample group. Characteristics of micronutrient supplement users *versus* non-users were compared using one-way ANOVA (for continuous variables) and chi square analysis (for categorical variables). Significance was set at *p* < 0.05 for analysis.

## 3. Results

**Figure 1:**
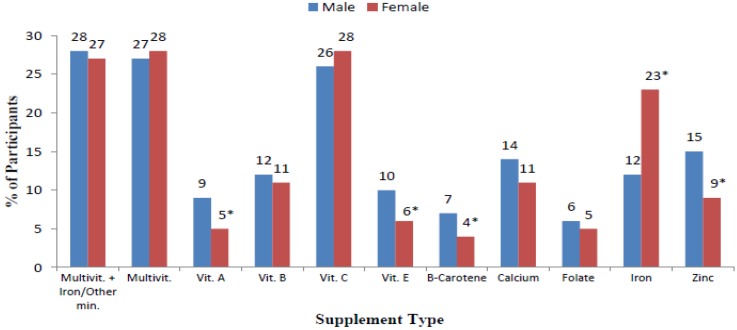
Sex differences between types of supplements used (*n* = 1302). * Denotes significant association (*p* ≤ 0.05) between sex and supplement use—chi square test.

Table 1 shows the demographic characteristics of participants and the association of these characteristics with supplement use. Overall, 79% of participants were female with a mean ± SD age of 20.5 ± 4.5 years and mean ± SD BMI of 22 ± 3kg/m^2^. The majority of participants (85%) were of Australian nationality and had mothers who had a year 12 education or less (41%). A total of 56% of participants reported the use of some type of micronutrient supplement as regularly as once a month or more. No significant association was observed between supplement use and age, BMI, nationality and maternal education. The only significant demographic characteristic associated with supplement use was sex (*p* < 0.001) with females (56%) more likely to consume supplements compared with men (44%). Males were more likely to consume vitamin A (*p* = 0.006), vitamin E (*p* = 0.046), zinc (*p* = 0.013) and β-carotene (*p* = 0.013) supplements, and females were more likely to consume iron (*p* < 0.001) supplements (Figure 1).

**Table 1:** Demographic Characteristics of participants and their association with supplement use.

Demographic	Total	Non-User	User	*p*^ a^
Total (*n*)	1306	571 (44)	731 (56)	
*Sex*				
Female (*n*(%))	1026 (79)	422 (75)	601 (83)	<0.001
Male (*n*(%))	267 (21)	144 (25)	122 (17)	
*Age (Years)*				
Mean (SD)	20.5 (4.5)	20.4 (4.3)	20.7 (4.7)	0.212 ^b^
17–19 (*n*(%))	769 (59)	340 (60)	426 (59)	
20–25 (*n*(%))	421 (33)	188 (33)	233 (32)	0.310
≥26 (*n*(%))	107 (8)	39 (7)	67 (9)	
*BMI*				
Mean (SD)	22.3 (3.2)	22.4 (3.1)	22.3 (3.2)	0.758 ^b^
Underweight (<18.5kg/m^2^) (*n*(%))	75 (6)	35 (7)	40 (6)	
Healthy (18.5–24.9 kg/m^2^) (*n*(%))	929 (78)	400 (77)	527 (79)	
Overweight (25–29.9 kg/m^2^) (*n*(%))	160 (14)	74 (14)	86 (13)	0.849
Obese (≥30) (*n*(%))	27 (2)	12 (2)	15 (2)	
*Nationality*				
Australian (*n*(%))	1090 (85)	477 (84)	610 (86)	
Asian (*n*(%))	98 (8)	47 (8)	50 (7)	0.556
Other (*n*(%))	90 (7)	43 (8)	47 (7)	
*Maternal Education*				
Year 12 or less (*n*(%))	499 (41)	229 (42)	269 (39)	
Trade/Apprenticeship or Certificate/Diploma (*n*(%))	271 (22)	116 (22)	155 (23)	0.565
University Degree or Higher (*n*(%))	457 (37)	195 (36)	259 (38)	

^a^ *p* values determined using chi square for categorical variables and; ^b^ One-Way Anova for continuous variables; SD = Standard Deviation.

**Figure 2:**
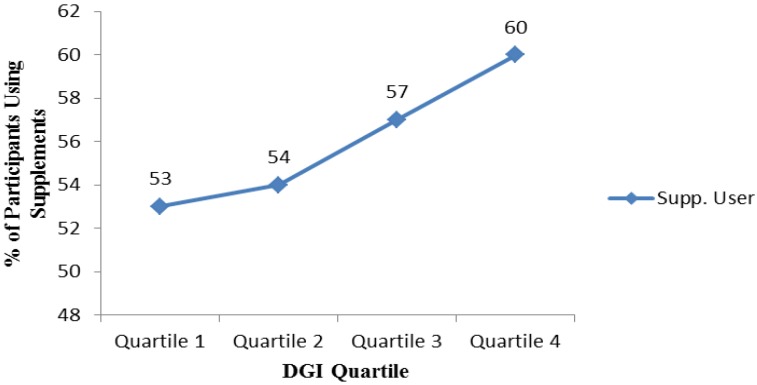
Percentage of supplement users per dietary guideline index (DGI) quartile (*n* = 1302). *p* = 0.019.

The association of health behaviour characteristics with supplement use is shown in Table 2. The majority of participants did not smoke (97%), consumed alcohol (84%), perceived their diet to be healthy or very healthy (90%) and used their peers as their main source of nutrition information (76%). Only 9% reported following a vegetarian or vegan diet, 11% followed a yeast, gluten or lactose free diet and 35% followed a low fat, low sugar or diabetic diet. No significant association was found between supplement use and smoking or alcohol consumption. Supplement users were significantly more likely (*p* = 0.009) to receive information from health professionals (11%) than non-users (7%). Supplement users were also more likely to be following a vegetarian/vegan diet (*p* = 0.013), low fat/low sugar/diabetic diet (*p* < 0.001), yeast/gluten/lactose free diet (*p* < 0.001) and to perceive their diet as healthy/very healthy (*p* = 0.001), compared with non-users. Overall the mean ± SD DGI score was 107 ± 18 and supplement users had a higher mean DGI score (*p* = 0.001) or were in a higher quartile for DGI score (*p* = 0.019) compared to non-users. The percentage of supplement users steadily increased as DGI quartile increased; 60% of participants within the highest quartile consumed supplements compared to 53% in the lowest DGI quartile (*p* = 0.019) (Figure 2).

**Table 2:** Health behaviour characteristics of participants and their association with supplement use.

Health Behaviour	Total	Non-User	User	*p*^ a^
Total (*n*)	1306	571 (44)	731 (56)	
Smoking				
Yes (*n*(%))	41 (3)	15 (3)	26 (4)	0.346
No (*n*(%))	1263 (97)	554 (97)	705 (96)	
Alcohol				
Yes (*n*(%))	1091 (84)	480 (84)	607 (83)	0.608
No (*n*(%))	213 (16)	90 (16)	123 (17)	
Perceived Health of Diet				
Very Unhealthy/Unhealthy (*n*(%))	127 (10)	72 (13)	54 (8)	0.001
Healthy/Very Healthy (*n*(%))	1132 (90)	478 (87)	651 (92)	
Source of Information On Food, Nutrition and Health				
Health Professionals				
Yes (*n*(%))	120 (9)	39 (7)	81 (11)	0.009
No (*n*(%))	1164 (91)	521 (93)	639 (89)	
General Public				
Yes (*n*(%))	502 (39)	214 (38)	286 (40)	0.632
No (*n*(%))	781 (61)	344 (62)	435 (60)	
Peer Group				
Yes (*n*(%))	980 (76)	439 (78)	538 (75)	0.180
No (*n*(%))	309 (24)	125 (22)	183 (25)	
Follow a Specific Diet				
Vegetarian/Vegan				
Yes (*n*(%))	103 (9)	34 (7)	69 (11)	0.013
No (*n*(%))	1065 (91)	485 (93)	576 (91)	
Low Fat/Low Sugar/Diabetic				
Yes (*n*(%))	484 (40)	170 (32)	311 (46)	<0.001
No (*n*(%))	738 (60)	364 (68)	373 (54)	
Yeast/Gluten/Lactose Free				
Yes (*n*(%))	132 (11)	37 (7)	95 (15)	<0.001
No (*n*(%))	1031 (89)	476 (93)	551 (85)	
Dietary Guideline Index (DGI)				
Mean (SD)	107 (18)	105 (18)	109 (17)	0.001^b^
Quartile 1: 19.9–95.1 (*n*(%))	326 (25)	158 (28)	167 (23)	
Quartile 2: 95.1–108.9 (*n*(%))	327 (25)	153 (27)	171 (23)	0.019
Quartile 3: 108.9–120.6 (*n*(%))	327 (25)	137 (24)	190 (26)	
Quartile 4: 120.6–141.7 (*n*(%))	326 (25)	123 (21)	203 (28)	

^a^ *p* values determined using chi square for categorical variables and ^b^ One-Way Anova for continuous variables; SD = Standard Deviation.

**Figure 3:**
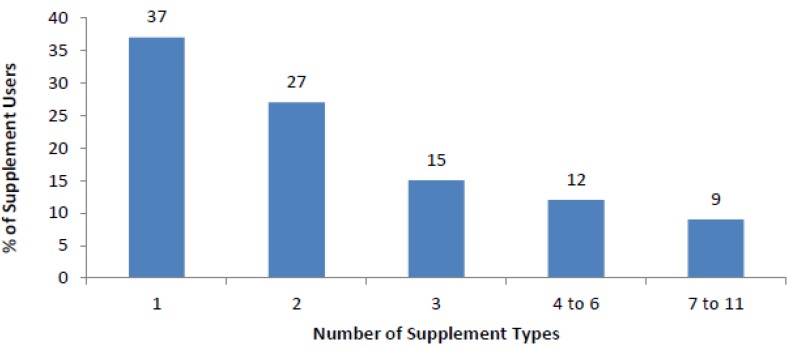
Number of different types of supplements used (*n* = 731).

Figure 3 shows the percentage of supplement users who consumed multiple supplement types. The majority of supplement users consumed more than one supplement (63%). Of the participants who reported to be a supplement user approximately 2.8 different supplements were consumed per participant. Multivitamin with Iron/other mineral (27%), multivitamin (28%) and vitamin C (28%) were the highest reported supplements consumed, iron was the most popular single mineral supplement consumed (20%). Supplements were usually taken once a day by 49% of supplement users, an equal percentage of supplements were reported to be consumed weekly (23%) compared to monthly (23%), and only a small percentage of supplements were consumed multiple times per day (5%).

## 4. Discussion

### 4.1. Overview

Our results indicate that micronutrient supplement use is common in this sample of university students with over half the participants reported consuming some type of vitamin and mineral supplement. The participants in the current study appeared to engage in healthy behaviours with the majority of the participants having a healthy BMI, being non- smokers, having a high diet quality as indicated by the average DGI score and perceived their diet to be healthy. Despite this, the high prevalence of supplement use indicates that many participants may not consider their dietary intake alone sufficient to meet their nutrient needs. The concept that dietary and nutrient adequacy can be achieved by “enjoying a wide variety of nutritious foods” as recommended in the Australian Dietary Guidelines [2] may not be well understood within this apparently health conscious population. A higher diet quality, as indicated by a higher DGI score, was positively associated with micronutrient supplement use, indicating that those consuming supplements are more likely to have a dietary intake consistent with dietary guidelines and therefore are more likely to be meeting their nutrient needs from diet alone.

With approximately two thirds of supplement users reporting the consumption of more than one type of supplement, one tenth consuming more than seven types of supplements and the majority consuming supplements daily, micronutrient intakes may be in excess of the recognised Upper Limit and have the potential to pose a negative health risk [1]. However, it is noted that some people may benefit from supplement use such as those with a nutrient deficiency [1], as well as pregnant or lactating women [5,6]. It was not possible within the current study to identify participants that may have benefited from supplement use as this data was not collected in the questionnaires, therefore we are unable to determine if participants’ supplement consumption coincides with current recommendations. Individuals consuming diets that exclude food groups may be at an increased risk of inadequate dietary intake [1,18,20], however within the current study only a small portion of participants reported to be following such diets. Those that did report following diets that exclude food groups were significantly more likely to consume micronutrient supplements, which may be appropriate as they are identified as an at risk group [1,2]. It is interesting to note that receiving information on food, nutrition and health from health professionals such as doctors, nurses, nutritionists and dietitians was also significantly positively associated with supplement use. This is an observation worth exploring as past research has indicated that 97% of U.S. dietitians recommended dietary supplements to their clients [19], and it may therefore be that health professionals are promoting supplement consumption. As many of the participants in this study may go on to become health professionals it is possible they may need to be further educated on the recommendation to meet nutrient needs from diet alone, as well as the lack of evidence supporting the use of micronutrient supplements to improve health and reduce the risk of disease [1,2,3].

### 4.2. Prevalence and Trends of Supplement Use: Comparison with Australian and US Population

The current study observed consumption of supplements at rates which are much higher than results from the Australian 2011–2012 NNPAS. The NNPAS observed 22% of the general population reported consuming micronutrient supplements and only 12% of 14–18 years and 17% of 19–30 years reported consuming supplements [8]. However, the 2011–2012 NNPAS data available on micronutrient supplement use were collected using one 24 h dietary recall [8], potentially a day where supplements were not consumed. Conversely, the 2003–2006 NHANES data on micronutrient supplement use were collected using a questionnaire that examined the participants supplement use over the past 30 days, similar to the current study [10]. These data revealed that 33% of the U.S. population used micronutrient supplements, 16% of those aged 14–18 years and 27% of those aged 19–30 [10], still much lower than the results of the current study. However, the NHANES results indicate that participants who had more than a high-school education were prevalent supplement users with 61% consuming supplements [10]. This suggests education status may play a significant role in supplement use and may explain the relatively high prevalence of supplement use in the current study. Furthermore, data from the 2003 to 2006 NHANES indicate that within the US population the majority of participants only consumed one type of dietary supplement (54%) and none consumed more than four, much less than the current study [10]. Although the U.S. population consumed fewer supplement types they were consumed more frequently with 79% of U.S. participants consuming supplements daily [10].

The most popular micronutrient supplement consumed by participants in the current study were multivitamins with iron or other minerals (27%), multivitamins (28%) and vitamin C (28%). These results were similar when compared with the sales data produced by the market research firm Euromonitor International Ltd on Australian dietary supplement sales [7] and the results from the 2011 to 2012 NNPAS [8], whereby multivitamins and vitamin C were the most popular supplements. Furthermore, both US and Australian population data found that a higher proportion of females consumed each micronutrient supplement compared to men [8,10], however this was not seen in the current study where men were more likely to consume vitamin A, E, β-carotene and zinc, and females were only significantly more likely to consume iron. In the 2011–2012 NNPAS use of iron supplements was not reported, however “other minerals” were reported to be used by only 0.5% of the overall population, suggesting that iron use is minimal [8]. It is unclear why iron use is so prevalent in the current female participants compared to the general population, however it may be due to the high proportion whom understand the increased risk of iron deficiency in premenopausal females [1,36].

### 4.3. Characteristics of Supplement Users

The only demographic characteristic associated with supplement use in the current study was sex, which was consistent with results from both the 2011–2012 NNPAS [8] and 2003–2006 NHANES [10]. Data from the 2011–2012 NNPAS and 2003–2006 NHANES found that micronutrient supplement use generally increased with age [8,10], this association was not seen in the current study, most likely due to the lack of diversity in age ranges. This lack in diversity also may be the reason for the absence of significant differences in supplement use with regards to nationality as US studies such as the 2003–2006 NHANES and 2007 children’s National Health Interview Survey (NHIS) found that Caucasians and Asian nationalities were more likely to consume supplements compared to other nationalities [10,12]. However, as this association was not seen in recent large population based Australian study [14] there is the potential for nationality to have a limited impact on supplement use within Australia. There was also a lack of diversity in participants’ BMI which again may have resulted in the absence of a significant association with micronutrient supplement use. Results from the 2007 ANCNPAS found no association for BMI and supplement use for children and adolescents, however US data from the 2007–2010 NHANES found a significant difference in supplement use between BMI ranges for those ≥20 years of age, with participants who had a healthy BMI more likely to consume supplements [11]. Both the 2007 ANCNPAS and 2007 NHIS found significant differences with regards to parental education and subsequent supplement use, where a higher education level was associated with a higher proportion of children consuming supplements [9,12]. However due to the majority of participants being older in the current study, parent influence on dietary intakes is most likely less prominent and may account for the lack of significant finding.

The association between health behaviours and micronutrient supplement use is not yet available for the Australian 2011–2012 NNPAS and therefore comparisons with the current study to the Australian population are difficult. However U.S data from the 2007–2010 NHANES on lifestyle factors associated with supplement use found that smoking status, perceived health status and alcohol consumption were significantly associated with micronutrient supplement use [11]. In the current study both smoking and alcohol were not found to be associated with supplement use, again this may be attributed to the lack of diversity for these variables. Perceived health of diet was also associated with micronutrient supplement use in the current study; those that perceived their diet as healthy or very healthy were more likely to consume micronutrient supplements compared to those who perceived their diet as unhealthy or very unhealthy. Out of health professionals, general public and peer groups, receiving information from health professionals was the only behaviour that was significantly associated with an increased likelihood of consuming micronutrient supplements. This was consistent with results from the 2007 to 2010 NHANES which revealed that almost a quarter of all dietary supplements (including non-micronutrient supplements) were reported to be used based on the advice of a health care professional [11]. Participants who followed a special diet were significantly more likely to consume supplements in the current study, this variable was not reported in the large nationally representative studies previously mentioned. However, one small study on 50 vegetarian and 24 omnivore females observed that none of the omnivore participants consumed supplements however 14% of vegetarians did [37]. Although, few studies assess the link between special diet statuses and supplement use, many studies address the fact that those on vegetarian, vegan, gluten free, lactose free diets may benefit from supplement use [1,20,21,38,39,40,41]. Finally, DGI score was found to be significantly associated with supplement use, both mean and categorical quartile results showed that those with a higher DGI score were more likely to consume micronutrient supplements. There are no other studies that compare DGI with micronutrient supplement use, however there are some studies that compare diet quality by assessing nutrient intakes with Estimated Average Requirement cut off points or other forms of diet quality analysis [13,15,16,17]. Studies conducted on Australian adolescents [15], 2007–2008 NHANES results [13], US college students [16] and the elderly [17] all were able to conclude that individuals consuming a diet more closely aligned with food and nutrient recommendations were more likely to use micronutrient supplements.

### 4.4. Limitations

A strength of this study was the large number of participants, however the lack of participant diversity in some demographic or health behaviour characteristics may have resulted in a lack of statistical power to determine the association with micronutrient supplement use. Furthermore, there were differences between the current study population and the general Australian population. Participants of the current study were younger, a higher proportion female, had a lower BMI, were less likely to smoke and had a higher diet quality when compared to previous Australian population based studies [8,35]. Therefore, caution should be taken in applying the results of the current study to the greater population.

In addition, the FFQ used in this study has not been updated since 1995 and therefore may not adequately represent the dietary patterns of the current population. Furthermore, the dietary supplements recorded were limited to that of the FFQ and resulted in some important supplements such as vitamin D or omega-3 fatty acids not being included. Data were collected via self-report, and participants may have under-, over- or mis-reported some information. Within this study it was not possible to determine how many participants would require supplements to either correct a defined nutrient deficiency or due to increased requirements such as pregnancy. Thus it is not possible to definitively determine if the high supplement use in this population group was aligned with current recommendations from national health agencies.

## 5. Conclusions

This study set out to identify the demographic, health behaviour characteristics and diet quality associated with micronutrient supplement use in a sample of university students undertaking studies for a food and nutrition degree. Micronutrient supplement use was prevalent in this study population and was associated with health behaviours that could be considered to indicate more health conscious individuals. Further research into what prompts health conscious individuals to consume micronutrient supplements is warranted. The results of this study further reinforce the notion that supplement users are already consuming diets aligned with public health guidelines. Further education needs to be centered towards the importance of meeting nutrient adequacy through diet alone.
